# Nonylphenol in Human Breast Milk in Relation to Sociodemographic Variables, Diet, Obstetrics Histories and Lifestyle Habits in a Turkish Population

**Published:** 2017-04

**Authors:** Sengul SISE, Cevdet UGUZ

**Affiliations:** 1. Dept. of Midwifery, Faculty of Health Sciences, Suleyman Demirel University, Isparta, Turkey; 2. Dept. of Medical Biology and Genetics, Faculty of Veterinary Medicine, Afyon Kocatepe University, Afyonkarahisar, Turkey

**Keywords:** Nonylphenol, Exposure, Turkish population, Human milk, Public health

## Abstract

**Background:**

Human breast milk is the most important food for infants and one of the main roads to exposure to toxic substances. In this study, nonylphenol (NP) levels in human milk samples collected from Turkish mothers (n=100) were determined, and the factors including mothers’ demographics, eating habits, obstetric histories, and usage of cleaning and cosmetic products were examined.

**Methods:**

Participants were mothers of randomly selected infants and toddlers from the Primary Health Care Center Number-8 in Afyonkarahisar City in western Turkey. The concentrations of NP in milk samples were measured using high-performance liquid chromatography (HPLC).

**Results:**

All the analyzed samples showed the occurrence of NP at levels up to 47 ng/ml. The mean± SE and the median NP concentrations were 10.1±0.98 ng/ml and 8.46 ng/ml or ppb, respectively. A negative correlation with infant age was observed. There was a significant correlation between fresh fish consumption and the level of NP in the breast milk of mothers. No significant association between body mass index (BMI) and the NP level in human milk of mothers was observed. The mothers who were using excessive cleaning products in comparison to those using less had significantly higher NP in their breast milk.

**Conclusion:**

This study provides the first report about NP levels in a population and characterizes individual variation, thus giving a measure of exposed infants through breastfeeding in Turkey.

## Introduction

Alkylphenol polyethoxylated (APEs) and their derivatives, such as nonylphenol ethoxylates (NPEOs) and octylphenol ethoxylates (OPEOs), have been used in many industrial and consumer applications including detergents, cosmetic products, and textiles (1). Their release into the aquatic environment has led to NPEOs everywhere in the environment. NPEOs are known to be highly toxic to aquatic organisms and are degraded into more environmentally persistent 4-nonylphenol (NP) when released into the environment.

NPEOs and NP act as endocrine disruptors in humans and animals (2). These substances interfere with hormones by binding to estrogenic receptors or influencing signal transduction or other cellular activities that induce estrogen-like action. Examples of hormonal disruption in male fish include synthesis of a female-specific protein (vitellogenin), effects on testicular growth and sperm counts, and intersex conditions (3).

NP can enter aquatic ecosystems through industrial and domestic wastewater flows and urban or agricultural runoff. Thus, it has been observed in river water, soil, sediments, and aquatic as well as terrestrial biota in many countries (3, 4). NP concentration in the aquatic environment has been shown to be as high as 300 ng/ml (5).

The consumption of food and drinking water are the main routes of human exposure to NP (6). NP was ubiquitous in different kinds of foodstuffs, for example, in Germany (7, 8) and in Sweden (9). Polyvinyl chloride (PVC) films are the sources of NP and widely used to package foods. PVC films contain NP that can migrate into the foods (10). NP has been observed in beverages and bottled water in China (11), vegetables and fruits in Taiwan (12), animal tissues such as fish and chicken (13), milk and egg samples (14), seafood (15), and drinking water and spring water (16). NP was present with an amount of 20 ng/g in fruit and vegetables (9). The mean of human exposures is around 27 μg NP/day (9). Therefore, it is necessary to monitor NP levels in biological samples for the risk assessment of this chemical.

Infants are more sensitive to endocrine disrupting chemicals than adults are, although a number of chemicals in an exposure remain equal. The main source of NP exposure for babies and infants is breastfeeding. Due to a large amount of fat in milk and the lipophilic character of APEs and their metabolites, the possibility of finding these compounds in human milk is considerably higher. Therefore, human milk has been widely used as a bioindicator to assess the extent of human exposure to these pollutants via various exposure routes.

NP has been detected in adult urine in the US (17) and in women’s adipose tissue in Spain (18). During the past decade, a few investigations regarding NP contamination in human milk have been conducted in various countries with a view to assessing the health risk for newborns and infants (7, 19–23).

Finding the factors affecting the NP presence in human milk is a complicated problem. It is not yet fully understood in what ways NP is bioaccumulated in the body. There has been no study on the concentration of NP in human milk in Turkey. In this work, 100 milk samples were collected from mothers living in Afyonkarahisar, and the concentrations of NP in these samples were examined. Infants’ daily intake of NP was estimated and compared to those in developed countries.

## Materials and Methods

### Chemicals and reagents

The technical grade nonylphenol 4-NP (CAS No: 84852-15-3), which contains a mixture of different isomers, was purchased from Sigma Chemical Co. (St. Louis, MO, USA). Acetonitrile and methanol are HPLC-grade and purchased from Merck (Darmstadt, Germany). The water used in the experiment was prepared from distilled water in our laboratory.

### Sample collection and storage

Participants were mothers of randomly selected infants and toddlers from the Primary Health Care Center Number-8 in Afyonkarahisar City in western Turkey. One hundred twelve mothers who came to the health center for baby vaccinations or PKU scanning between Jan and Apr 2010 were involved in this study and asked to participate by completing a questionnaire. All mothers were breastfeeding at the time of the study. The milk samples were collected manually between mid-morning and noon during breastfeeding either by the subjects themselves or with the assistance of nurses. Inclusion criteria specified that the target volume was at least 10 ml from each mother during one sampling, and the age of infants and toddlers was ≤20 months. Only 100 participants provided required milk samples. First, they collected milk by manual expression prior to the midday feeding of their infant (foremilk). After this, they collected milk by manual expression (hind-milk). Since the fat content of hindmilk is higher than that of foremilk, it was used in the analysis. The samples were collected in chemical-free glass tubes. Plastics were excluded throughout the analytic procedure to avoid further contamination. All the samples were frozen immediately after collection and stored at –21 °C until chemical analysis.

The research protocol was approved by the Ethics Committee of the Ministry of Health in Ankara (document number: 2010/178708), and an appropriate written informed consent was obtained from all women.

All mothers were required to answer a questionnaire to provide sociodemographic data, obstetrics information, and other information such as consumption and dietary habits, use of packaging materials, cleaning products and cosmetics.

### Sample preparation and instrumental analysis

The samples were incubated at 60 ^o^C for 10 min; 2 mL of acetonitrile was added and fully mixed. The mixture was consequently centrifuged (1500 rpm, 5 min) at room temperature, and the supernatant of 5.0 ml was transferred to a glass tube. After evaporation, the samples were reconstituted with 100 μl of acetonitrile, centrifuged (1500 rpm, 10 min). Then, 20 uL of the supernatant was injected for HPLC analysis. NP levels were determined by HPLC (Agilent 1100 series, Munich, Germany). The chromatographic column used for analysis was a Macherey-Nagel C18 ODS (240 mm x 4.6 mm I.D., 5 μm). The C18 SPE cartridges (Agilent SampliQ, USA) were also used in the experiments.

The HPLC determination of NP was performed using a mobile phase: solvent A: acetonitrile and solvent B: water. The flow rate was 1.0 ml/min at 25 °C. The sample injection volume was 20 μl. The system was run in a linear gradient: 0–6.0 min A-70%, B-30%, 6.0–6.2 min mobile phase An increased from 70% to 100%, held for 6.8 min, after 13 min A-70%, B-30% and stopped at 18 min (24). Using fluorescence detection (λ_ex_=227 nm and λ_em_=313 nm), NP has an apparent retention time of 7.7 min. The concentrations of 4-NP in human milk were obtained from the corresponding internal calibration plots. A very satisfying linearity was obtained between peak areas and the corresponding standard concentrations of NP ranging (n=5) from 0.01 to 100 μg/mL, and the coefficient of determination (R^2^) obtained by regression analysis was higher than 0.99. The limit of detection (LOD) was 0.26 ng/ml. Recoveries were determined by five to replicate analyses of the sample, spiked with known concentrations of NP. The average recoveries were between 81% and 92%, and the relative standard deviations (RSD) were 5% and 8%, respectively. Moreover, procedural blanks were analyzed for quality control and all blanks showed the values smaller than the LOD value. The chemical name 4-NP is common to the entire group of isomers of NP (550 isomers) with the alkyl chain in para position. The chain can be either linear or branched. HPLC analysis cannot discriminate different isomers in the mixture and they resolve in the chromatogram with a unique peak. This means that the results of the study express the total concentration of NP isomers in the human milk samples analyzed.

### Analysis of data

Data were analyzed using SPSS ver.17 (Chicago, IL, USA). The Shapiro-Wilk’s test was used to test the normality, and the data analyzed were not normally distributed. Therefore, differences in the concentrations of NP between different groups were determined with the non-parametric tests (Mann-Whitney U and Kruskal-Wallis H). Spearman rank correlations were also used to investigate the relationship between NP concentrations and socio-demographic and maternal parameters. The significance level was set at two-sided *P*≤0.05. All values are reported as means with their standard errors unless otherwise stated.

## Results

### Characteristics of the participants

Analysis of data showed that the age of the mothers participating in this study ranged from 18 to 40 yr old (Table 1). The mean age of mothers ± SE was 25.9 ± 0.45 yr, and mean body mass index (BMI) was 31.1 ± 0.59 kg/m^2^ at the time of the study. There were mostly housewives (93.0%) in the sample, and there was a small proportion of mothers with a high school or higher education (22.0%).

**Table 1:** Characteristics of the mothers (mean±S.E. or %)

**Characteristics**	**Mean±S.E. or %**
Age of mothers (yr)	25.9 ± 0.45
BMI after pregnancy (kg/m^2^)	31.1 ± 0.59
Age of babies (months)	10.97 ± 0.63
Number of pregnancy	2.4 ± 0.12
First menstruation age	13.3 ± 0.11
Number of children	2.39 ± 0.12
1	27.0
2	33.0
>3	40.0
Maternal education	
< High school	78.0
≥ High school	22.0
Occupation	
Housewives	93.0
Workers	7.0
Place of residence	
Far from industry	89.0
Close to industry	11.0
Number of meals in a day	
2	24.0
3 or more	76.0
Drinking water	
Tap	63.0
Bottled	27.0
Well	10.0

Most of the mothers (89.0%) lived in a residential environment far from industry. Among obstetric information, the mean of first menstruation age was 13.3 ± 0.11 (range: 10–16). A proportion of 40.0% had more than two children, with a mean number of 2.39 ± 0.12 (range: 1–6). Forty percent of infants were ≤ 6 months old and the mean infant age was 11.0 ± 0.63 (range: 0–20). In our study, most of the mothers (76.0%) ate more than three meals a day. The frequencies of mothers who consumed fish and meat 2–3 times a week were 46.0% and 41.0%, respectively. Tap water (63.0%) was used mainly for drinking in the present sample, followed by bottled water (27.0%) and well water (10.0%).

### NP concentration in human milk

Table 2 shows the characteristic information of the 100 participants involved in this study and the level of NP concentration in human milk. Only some of the significant results were shown for clarity.

**Table 2:** Mean concentration of nonylphenol in human milk according to characteristics of the mothers

**Variables**	**n=100 (%)**	**NP (ng/ml) Mean± SE**	***P*-value**
**Infant age**			
≤6 months	40.0	18.1±1.56	0.000
>6 months	60.0	4.80±0.66	
**BMI**			
<30 kg/m^2^	47.0	10.9±1.47	0.344
≥30 kg/m^2^	53.0	9.40±1.32	
**Fish consumption^a^**			
Frequently	46.0	14.11±1.52	0.000
Not frequently	54.0	6.69±1.08	
**Exposure to cleaning products^b^**			
Frequently	64.0	11.80±1.22	0.002
Not frequently	36.0	7.10±1.55	

a *Frequently*: once or twice a week; *Not frequently*: very seldom or once or twice a month.

b *Frequently*: once or twice a week; *Not frequently*: once or twice a month.

The highest amount NP measured was 47.5 ng/ml. The geometric means of NP concentrations were 5.01 ng/ml. The mean ± SE and median of NP concentrations were 10.1 ± 0.98 ng/ ml and 8.46 ng/ ml, respectively.

There was no significant relationship between the NP concentrations and the mothers’ age (r = −0.048, *P*=0.636), and the current BMI (r = −0.188, *P*=0.062). The mean NP levels increased with the infant ages (*P*=0.0001) and showed an inverse association (r= −0.780, *P*=0.0001). No correlations were observed between the NP concentrations in human milk, the first menstruation age (r=0.084, *P*=0.408), and the number of pregnancies (r=0.025, *P*=0.805).

The NP levels in human milk of mothers who declared eating fish at least once or twice a week appeared higher in comparison to samples from mothers who ate fish only once or twice a month or seldom. This trend was statistically significant (*P*=0.0001). No correlation was found between the food packaging materials and NP levels in human milk.

There is a significant correlation between the frequency of exposure to cleaning products used for cleaning tasks and NP levels in human milk (*P*<0.01). A majority of participants declared using perfume, deodorant, roll-on, but they did not acquire NP in their milk. Although skin cream seemed to contribute NP accumulation compared to perfume, deodorant or roll-on, its contribution for NP accumulation in human milk was not significant (*P*>0.05). Overall, the use of cosmetics did not cause a significant increase in the breast milk NP levels of mothers.

## Discussion

NP is found in foods and water in ng to mg amounts (9). Moreover, NP is also abundant in household air and dust (25). Exposure may occur from food consumption and drinking water as well as the inhalation of contaminated air and dust particles.

The lipophilic character of NP, as well as octylphenol (OP), implies that these substances are highly resistant to biodegradation in the environment and will bioconcentrate or bioaccumulate in the fatty tissues of fish (26) and terrestrial organisms (3). Once NP enters into the sediment, the half-life of degradation is estimated to be approximately 60 yr (27). The current NP presence in the environment will continue to exert its adverse effects on living organism for the next sixty years.

In our work, the concentrations of NP in breast milk of 100 mothers were measured and their potential routes of exposure were determined. Many factors can affect NP accumulation and distribution in the human body, including sociodemographic status, obstetric history, dietary habits, and use of cleaning and cosmetics products. Therefore, we investigated the relationship between NP concentrations and various factors, and we assessed potential NP sources in human samples.

There is no significant correlation between the NP levels in human milk and the age of mother, profession, education, BMI, or place of residence, in accordance with other studies (18, 23). Although the NP levels in adipose tissue were significantly negatively associated with BMI (18). There was no statistically significant relationship between the concentrations of NP in human milk and BMI (23). Environmental chemicals may disrupt homeostatic controls on adipogenesis and normal development. However, it is not clear whether exposure to NPs contributes to obesity. There is a correlation between the level of NP and the infant age. The mechanism of this correlation is obscured. In the literature, there were no differences in the contents of fat in human milk from 1 to 12 months of lactation (28). However, the decreasing of NP concentration in milk is probably linked to lactation, known to act as a detoxification process in mothers as it is the case for other toxicants.

Alkylphenols such as NP may not be detectable in water, but they can be detected in fish due to the bioconcentration (26, 29). NP levels were detected in freshwater fish in Turkish rivers, with the amounts of 0.11–0.59 μg/g in fish, although no NP was detected in water where they live (26). In addition, NP was concentrated in the sediment with the amounts between 3.15–4.46 μg/g, but not in the water (26). This may pose a potential danger to humans since demersal fish feeding on sediment may uptake NP, which finally ends up in human beings through the food chain. In fact, in Italy, 32 ng/ml NP has been measured in the milk of mothers who consumed fish frequently (3 times a week) (21). In accordance with these findings, there was a significant correlation between the NP levels in human milk and the consumption of fresh fish.

In this study, no correlations were found between NP levels and the frequency of meat consumption. NP levels showed a slight increase with diet plans and the number of meals, though neither of these proved statistically significant (*P*>0.05). In Germany, NP has been identified in different foodstuffs (fish, fruits, and vegetables) (7, 8). Concentrations were not apparently related to the fat content of the foods. This suggests that origin of the NPs in foods arises from multiple sources. One way of NP contamination to food is the food packaging process (10). Foods and drinking waters can be contaminated by NP, and humans can be exposed to this chemical through their consumption (6, 30). During long storage time, NP can migrate from packaging materials into foods or waters under high temperatures (31). However, we did not find any correlations between the NP levels and the use of plastic packaging materials for foods.

NPs can be found in household cleaning products including liquid detergents and all-purpose cleaners. They are also found in cosmetic products used for the skin, eye, face, and hair (cream, makeup, deodorant, bathing products, etc.) (3). NP exposure may occur through the skin from these products, used repeatedly and routinely at home to maintain cleanliness. This increases the chances for bioaccumulation of NP in the human body. We analyzed the potential influence on mean NP values of some practices of the mother such as use of cleaning products and wearing gloves as a precaution when cleaning. We have found a significant correlation between the concentration of NP and the exposure to cleaning products when they are used for cleaning. This suggests that NP is absorbed from the skin, which contributes to significant elevations of NP in human milk (*P*<0.01). However, no statistically significant relationship was observed between the level of NP and cosmetics use. We have found no other report on NP exposure from cosmetics products to compare with our data. The consumption of cosmetics in Turkey is very low due to low GDP per capita. In the USA or EU, 80% of the population uses cosmetic products. In Turkey, this ratio is about 20% (32). Socioeconomics is a major factor that affects cosmetics use of women in developing countries.

NP levels in human milk reported in different countries (Table 3).

**Table 3:** Mean concentration of nonylphenol (ng/g or ng/ml) in human milk from different countries

**Country**	**Germany**	**Japan**	**Italy**	**Taiwan**		**Turkey**
Mean	0.3	1.05	32	4.4	4.47	10.1
Range	-	0.65–1.40	13.4–56.3	1.70–11.6	-	0.26–47.5
Intake (ng/ml b.w./day)	0.05	0.17	5.04	0.69	0.71	1.59
n	1	3	10	19	59	100
References	Guenther et al. (7)	Otaka et al. (19)	Ademollo et al. (21)	Lin et al. (22)	Chen et al. (23)	This work

The NP levels measured in Turkey were 3.2 times lower than in Italy (21). However, NP levels measured in Turkey are 10 to 30 times higher than those from developed countries such as Japan and Germany, respectively. The differences in the levels of NP in different countries may be due to the culture, financial level, dietary habits as well as different levels of industrialization.

After having the concentrations of NP in human milk, one can calculate the infant’s daily intake of NP, which is more meaningful than the absolute contamination values. An infant with a body weight (b.w.) of 5 kg can drink milk of 788 ml per day (33). Using the NP concentrations of 10.1 ng/ml (mean) and 8.46 ng/ml (median), respectively, the intake would be 1.59 (mean) and 1.33 (median) ng/ml b.w./day, respectively. The tolerable daily intake was determined to be 5 ng/ml b.w. (34) and our results are lower than this value. The mean daily intakes for human milk were calculated as 0.05 ng/ml b.w./day in Germany (7), 0.17 ng/ml b.w./day in Japan (19), 5.04 ng/ml b.w./day in Italy (21), and 0.69–0.71 ng/ml b.w./day in Taiwan (22, 23). WHO suggests that mothers should breastfeed their babies for at least six months even though there is a risk posed by persistent organic pollutants in human milk (35).

## Conclusion

This is the first study investigating NP concentrations in human milk in Turkey. This study provides a baseline level for NP in human milk, besides, might reflect multiple exposure routes for the general Turkish population. The NP levels or daily intake was found to be higher than those of some countries were. NP contamination is high, and the people who live in this region are exposed to this chemical. We have found a significant correlation between the level of NP in human milk and the consumption of fresh fish. In addition, exposure to cleaning products significantly contributes to the NP levels. There is a need for further investigation and assessment of NP concentrations in human and various foodstuffs as well as the main routes of exposure in our population. In Turkey, there are no government regulations about the usage of NP or related chemicals. Since human milk is important for infants, it is essential to monitor the NP levels and to estimate the time trend of NP in the end.

## Ethical considerations

Ethical issues (including plagiarism, informed consent, misconduct, data fabrication and/or falsification, double publication and/or submission, redundancy, etc.) have been completely observed by the authors.
